# The effect of Hydrodistension in combination with Pentosan Polysulfate on treatment outcomes and compliance in the treatment of bladder pain syndrome

**DOI:** 10.12669/pjms.35.1.172

**Published:** 2019

**Authors:** Adnan Simsir, Fuat Kizilay, Ceyhun Ozyurt

**Affiliations:** 1 Dr. Adnan Simsir, Department of Urology, Ege University School of Medicine Izmir, Turkey; 2 Dr. Fuat Kizilay, FEBU, Department of Urology, Ege University School of Medicine Izmir, Turkey; 3 Dr. Ceyhun Ozyurt, Department of Urology, Ege University School of Medicine Izmir, Turkey

**Keywords:** Bladder Pain Syndrome, Interstitial Cystitis, Pentosan polysulfate, Hydrodistension

## Abstract

**Objective:**

In the present study, we investigated the efficacy of bladder hydrodistension combined with pentosan polysulfate (PPS) treatment in interstitial cystitis (IC)/bladder pain syndrome (BPS).

**Methods:**

In this study, 339 patients diagnosed with IC/BPS were categorized into two groups. The first group only received 300 mg/day PPS, while the second group received 300 mg/day PPS following bladder hydrodistension. The results were evaluated at the 3rd, 6th, and 12th months after the first dose using the interstitial cystitis symptom index (ICSI), international cystitis problem index (ICPI), visual analog scale (VAS), and female sexual function index (FSFI).

**Results:**

PPS treatment started just after hydrodistension was significantly more effective than PPS treatment alone and combined treatment significantly reduced the rate of non-compliance such that, at the end of the 3rd month, 12.1% patients in Group-1 did not continue their treatment whereas only 1.9% of patients in Group-2 did not continue.

**Conclusions:**

The study results indicate that PPS treatment started just after hydrodistension yields significantly better results in terms of both symptom improvement and treatment compliance in patients with IC/BPS.

## INTRODUCTION

Despite the advancements in medicine that have occurred at an unprecedented pace in the 21st century, there has not been a similar level of improvement in the treatment of interstitial cystitis/bladder pain syndrome (IC/BPS), causing continuing disappointment among both physicians and patients. Until 2002, the National Institute for Diabetes and Digestive and Kidney Disease (NIDDK) criteria were considered sufficient for the diagnosis.^1^ However, the International Continence Society proposed a new definition that year due to inconvenience of those criteria for routine use. Accordingly, IC/BPS was defined as “the complaint of suprapubic pain, related to bladder filling accompanied by other symptoms, such as increased daytime and nighttime frequency, in the absence of proven urinary infection and other obvious pathology of the lower urinary tract”.^2^-^5^

Epidemiological studies report high prevalence rates of IC/BPS in the population, ranging between 2.7-6.5%.^6^,^7^ The disease is significant in that it considerably affects patient’s life-quality by causing various problems such as ineffective social and work life, avoidance of sexual intercourse, and major depression.^8^

Unfortunately, for a disease of such high prevalence that causes significant impairment in life quality, no definitive treatment has yet been established. Pentosan polysulfate sodium (PPS), intravesical dimethyl sulphoxide (DMSO), and many oral agents are used despite a lack of FDA approval (e.g., antihistamines, tricyclic antidepressants, antispasmodics, and steroids), as well as hydrodistension, botulinum toxin injections, neuromodulation, and even cystectomy are methods currently used in treatment.^9^

The only therapeutic agent currently approved for oral treatment of IC/BPS is PPS.^10^ However, data in the literature as well as experience from daily practice suggest that this treatment is ineffective in some patients, and so there is still an ongoing search for alternative treatments for the disease.

In the present study, we compared the treatment success of oral PPS treatment alone and oral PPS treatment in combination with bladder hydrodistension for the purpose of enhancing treatment efficacy and treatment compliance in patients diagnosed with IC/BPS.

## METHODS

### Patients

The study was performed in a single center. We retrospectively reviewed 415 patients who were diagnosed with IC/BPS upon presenting to our center between November 2005 and July 2015. Since male lower urinary system pathophysiologies have different and more complicated presentations, the present study included only female patients in order to provide homogeneity in the study group. After applying study inclusion and exclusion criteria, the study included 339 patients. The inclusion criteria included female sex, patient aged 18 years old or older, complaints present for at least 6 months, no treatment received for IC during the last 6 months, no history of neurological disease, no history of previous pelvic surgery, no history of pelvic irradiation, not diagnosed with diabetes mellitus, no history of clinical urinary system infection within the last 1 month, and no pelvic organ prolapse. The exclusion criteria were consisted of patient younger than 18 years old, pregnancy or lactation, abnormal liver function tests, anticoagulant treatment, thrombocytopenia, hemophilia, history of gastrointestinal bleeding, and urolithiasis history.

The diagnosis was based on medical history, physical examination, urinary culture antibiogram, cystoscopy and biopsy. The symptoms had to be present for at least 6 months. For every patient, scores from interstitial cystitis symptom index (ICSI), interstitial cystitis problem index (ICPI), and visual analog scale (VAS) for assessment of pain were recorded both before and after treatment for comparison. Sexually active individuals were assessed with the female sexual function index (FSFI).

### Study Design

**Fig.1:**
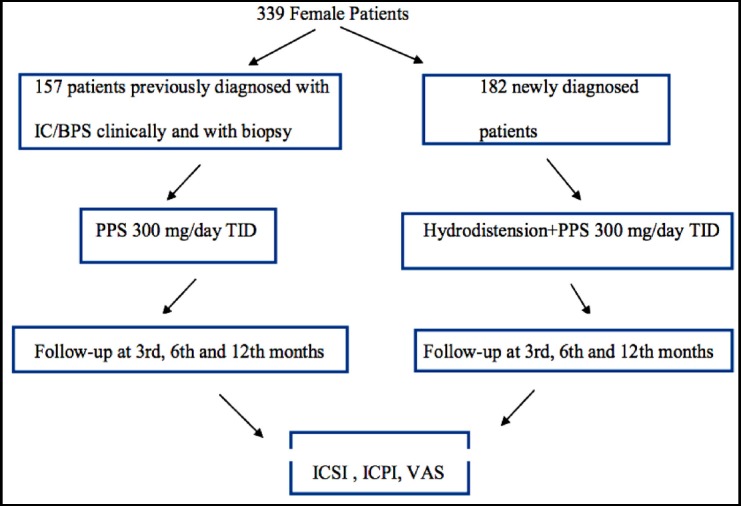
Study Design.

Patients were categorized into two groups: Group-1 comprised patients who were previously diagnosed based on medical history, urinary culture antibiogram, physical examination, cystoscopy, and biopsy findings but had not received any treatment apart from analgesics within the last 6 months, whereas Group-2 comprised patients who presented for the first time and were newly diagnosed. In addition to hydrodistension and biopsy performed for the initial diagnosis of all these patients in Group-2, other atypical findings were also noted and treated (e.g., Hunner’s ulcer). The main purpose here was to avoid additional morbidity caused by repeating the same procedures in patients already diagnosed with biopsy and cystoscopy. In the first group, oral PPS treatment was initiated at 300 mg/day. In the second group, oral PPS treatment was initiated as early as possible after confirming the diagnosis with cystoscopy, hydrodistension, and biopsy. Patients were evaluated at the 3rd, 6^th^, and 12th months after initiation of PPS treatment using the same assessment methods (Fig.1). The study was conducted in accordance with the Helsinki declaration, and the hospital’s ethics committee approved the study.

### Statistical analysis

For statistical analysis of the data, Pearson chi-square test, Cochran-Mantel test and Pairwise comparison tests were used, and p<0.05 was accepted as statistically significant.

## RESULTS

After applying the study inclusion and exclusion criteria to the 415 patients reviewed retrospectively, 76 were excluded from the study. Thus, the study included a total of 339 patients. According to the patient follow-up records, 50 patients in Group-1 (31.8%) and 21 patients in Group-2 (11.3%) did not show up for the control visits at the end of the first year. Mean patient age for the whole study group was 42.8 (21-74) years. For all patients, the diagnosis was made according to the definition of ICS.^4^,^5^ The diagnosis was confirmed in every patient with cystoscopy findings and biopsy results. Among all patients, four patients were found to have Hunner’s ulcer. All of these 4 patients were in the second group and all of them underwent ulcer cauterization and hydrodistension immediately after the biopsy and PPS treatment was started. We did not include urodynamic findings in the analysis because urodynamic findings are not included in the ICS diagnostic criteria; also, urodynamic testing is not routinely performed in our center in order to avoid unnecessary invasive interventions.

Mean duration of symptoms was 14 months (range 8-48 months). Of the 339 patients included in the study, 268 (79.05%) patients regularly attended all control visits until the end of the first year. Based on patients’ assessment results at the end of the first year, PPS treatment at 300 mg/day resulted in quite good improvement of symptoms in patients from both groups (Table-I).

**Table-I:** Evaluation of the efficacy of PPS in study groups.

	Number of patients	Age	ICSI	ICPI	VAS
Prior to treatment	157	43.6(24-70)	12.2(7-16)	9.4(5-11)	6.7(4-9)
*Group-1*					
Control at 1st year	107	47.3(25-70)	7.2(0-10)	7.0(4-8)	5.1(0-10)
P		0.12	<0.001	0.02	<0.001
Prior to treatment	182	42.0(21-74)	15.1(6-19)	11.8(5-16)	7.8(3-10)
*Group-2*					
Control at 1st year	161	46.4(29-70)	6.5(0-11)	6.3(3-10)	4.8(0-7)
P		0.34	<0.001	<0.001	<0.001
Prior to treatment	339	42.8(21-74)	13.6(6-19)	10.6(5-16)	7.25(3-10)
*Total*					
Control at 1st year	268	46.8(25-70)	6.85(0-11)	6.65(3-10)	4.95(0-10)
P		0.43	<0.001	<0.001	<0.001

Group-I: PPS 300 mg TID without hydrodistension, Group-II: PPS 300 mg TID initiated early after hydrodistension

Regarding comparison of the groups at the pre-treatment phase, it was observed that patients in Group-1, who had been diagnosed previously and had received various treatments before, but none except analgesics within the last 6 months, had milder symptoms in comparison to Group-2. Additionally, comparison at the end of the 3rd month of treatment showed that although patients in Group-2 had more severe symptoms, they responded very well to the treatment, and that treatment success was higher in comparison to Group-1.

Another interesting finding was related to attendance to the control visits. Whereas 19 patients (12.1%) from Group-1 did not attend control visits at the end of the third month, this number was 2 (1.9%) in Group-2 (p<0.001). At the end of the sixth month, the number of patients who did not attend control visits was 13 (9.42%) in Group-1, and 4 (2.22%) in Group-2 (p<0.001). Comparison of the results at the end of the sixth month showed that both groups had similar treatment success rates.

During the last six months of the follow-up period (starting from the 6th month until the 12th month), 18 (14.4%) patients from Group-1 and 15 patients (8.52%) from Group-2 did not attend control visits (p<0.001). Regarding evaluation of the last six months treatment period, it was observed that both groups showed progressive improvement, and that there was no statistically significant difference between the groups. Table-II shows comparative evaluations between the groups. When the FSFI results of the patients were evaluated before and after treatment to evaluate their sexual activity, only 4 patients in group 1 and 3 patients in group 2 had sexual activation at the time of diagnosis. After treatment, this number increased to 6 in group 1 and 8 in group 2. However, when the sample size ratios were considered, 2.54% of the patients in group 1 and 1.64% of the patients in group 2 were sexually active, so it was decided that the rates of recovery of sexual activation with treatment would not be a reliable scientific anecdote.

**Table-II:** Comparison of the efficacy of PPS between patient groups.

	Group-1	Group-2	p
*Prior to treatment*			
Number of patients	157	182	
Age	43.6 (24-70)	42.0 (21-74)	0.34
ICSI	12.2 (7-16)	15.1 (6-19)	0.041
ICPI	9.4 (5-11)	11.8 (5-16)	0.046
VAS	6.7 (4-9)	7.8 (3-10)	0.048
*3^rd^ Month*			
Number of patients	138	180	
Age	44.3 (25-70)	41.65 (21-72)	0.18
ICSI	8.3 (5-13)	6.3 (4-14)	<0.001
ICPI	8.1 (5-9)	6.7 (4-14)	0.02
VAS	6.1 (1-7)	4.1 (1-8)	<0.001
*6^th^ Month*			
Number of patients	125	176	
Age	44.6 (25-70)	41.1(21-71)	0.21
ICSI	6.9 (6-9)	6.0 (4-12)	0.15
ICPI	7.1 (4-8)	6.3 (0-12)	0.09
VAS	4.2 (0-6)	4.9 (0-5)	0.08
*12^th^ Month*			
Number of patients	107	161	
Age	47.3 (25-70)	46.4 (29-70)	0.82
ICSI	7.2 (0-10)	6.5 (0-11)	0.21
ICPI	7.0 (4-8)	6.3 (3-10)	0.13
VAS	5.1 (0-10)	4.8 (0-7)	0.18

ICSI: Interstitial Cystitis Symptom Index, ICPI: Interstitial Cystitis Problem Index, VAS: Visual analogue scale.

## DISCUSSION

In the present study, we investigated whether hydrodistension caused any change in the efficacy of PPS treatment in 339 IC/BPS patients diagnosed according to ICS criteria and confirmed with cystoscopy and biopsy. First, we observed that PPS 300 mg/day TID was very effective in treatment of IC/BPS. Literature data indicate that, compared to placebo, PPS treatment results in greater than 50% more improvement in patient symptoms.^11^-^16^ On the other hand, some researchers reported that efficacy of PPS treatment dropped to nearly 10% in the long-term (average 18-24 months), thereby causing reduced patient satisfaction.^17^ By contrast, in a dose-adjustment study, Nickel et al. observed that 300 mg/day TID was the ideal dosage, and that as the duration of treatment increased, patient satisfaction increased and symptom severity decreased.^18^ Our results also clearly showed that increased treatment duration resulted in greater patient satisfaction and greater improvement in symptoms. Considering the drug’s mechanism of action, a certain amount of time is needed for mucosal repair and for restoration of the glycosaminoglycan (GAG) layer, and therefore, for improvement of symptoms. This required time varies from patient to patient, and it is difficult to specify an exact time period. Presence of such an interval was the main reason for conducting the present study. We designed the present study in order to investigate the potential benefit of combining the slowly acting PPS treatment with a rapid and immediately effective method with a low risk of complications that would be effective until the effects of PPS emerged.

Therefore, we used hydrodistension performed under general anesthesia for this purpose. AUA guidelines recommend hydrodistension as the third line treatment in BPS^19^ (Table-III). Bladder distention has long been used in diagnosis and treatment of IC/BPS.^20^ Particularly, high pressure (>80cm H_2_O) and prolonged (>10 min.) hydrodistension has been shown to be more beneficial; however, it should be noted that it has higher complication rates (bladder rupture, sepsis, hematuria), and therefore, must be performed with caution.^21^-^23^ Hydrodistension is known to be immediately effective in the treatment of IC/BPS. Thus, we observed in our study that hydrodistension caused relief of symptoms until the effects of PPS treatment could be seen. Indeed, at the end of the first three months, symptoms scores were significantly lower among patients who underwent hydrodistension just prior to initiation of PPS treatment. For that reason, we believe hydrodistension can be an effective treatment method as an adjunct to PPS.

**Table-III:** Treatment of painful bladder syndrome (2011 AUA guideline).

First Line:	General relaxation, pain management, patient education, and behavioral modification.
Second line:	Appropriate manual physical therapy technique. Oral Amitriptylline, Cimetidine, Hydroxyzine and PPS. Intravesical-DMSO, Heparin, Lidocaine, and pain management
Third line:	Cystoscopy under general anesthesia with hydrodistension, pain management, treatment of Hunner’s ulcer if present.
Fourth line:	Neuromodulation
Fifth line:	Cyclosporin, intradetrusor botulinum toxin and pain management
Sixth line:	Diversion with/without cystectomy, pain management and substitution cystoplasty

At the end of the first three months, the proportion of patients who did not attend control visits was significantly higher in Group-1. This may be because these patients could not wait for optimal treatment efficacy to appear, and went on search for an alternative physician or treatment method. The number of patients who did not show up for follow-up was quite low in the group who received hydrodistension in combination with PPS treatment.

Another study goal was to examine how IC/BPS affected female sexuality, and whether treatment could solve this problem. Based on evaluation of patients using the FSFI, it was observed that the majority of patients did not have a functional sexual life at all due to IC/BPS, and that no matter the level of improvement with treatment, patients continued an asexual life due to the pain they suffered during sexual intercourse.

## CONCLUSIONS

IC/BPS affects patients physically, but it can cause serious psychological, social, and sexual problems. PPS reduces the symptoms to nearly half, and is widely preferred due to the convenience of oral administration. The drug’s action is not immediately observed due to the nature of the disease, and it is a proven fact that its efficacy increases with continued use. Therefore, it would be appropriate to initiate PPS 300 mg/day treatment as early as possible after bladder hydrodistension under general anesthesia, as doing so would provide both acute symptom relief and increased patient compliance. Nevertheless, further studies and ideas related to combination of these two methods would aid in increasing the number of alternative treatment options.

### Authors’ Contribution

**AS, FK** conceived, designed and did statistical analysis & editing of manuscript.

**AS, FK** did data collection and manuscript writing.

**AS, FK and CO** did review and final approval of manuscript.
